# Association between early depressive symptoms after stroke and trajectories of functional recovery among patients with acute ischemic stroke: a longitudinal study

**DOI:** 10.3389/fneur.2026.1737884

**Published:** 2026-01-27

**Authors:** Fanfan Li, Xingjin Song, Cuicui Zhang, Chi Peng, Ting Hu, Xiue Wei, Liangqun Rong, Haiyan Liu

**Affiliations:** 1 Department of Neurology, Second Affiliated Hospital of Xuzhou Medical University, Xuzhou, China; 2 Graduate School of Xuzhou Medical University, Xuzhou, China; 3 Department of Neurology, Beijing Tiantan Hospital, Capital Medical University, Beijing, China; 4 Department of Emergency, Changhai Hospital, Naval Medical University, Shanghai, China

**Keywords:** acute ischemic stroke, depressive symptoms, functional recovery, stroke outcome, trajectories

## Abstract

**Background:**

Depressive symptoms are very common in the acute phase of stroke; however, its impact on distinct functional recovery trajectories in acute ischemic stroke (AIS) patients remains unclear. Our study aimed to depict the functional recovery trajectories within 6 months after stroke and explore the association of early depressive symptoms with these recovery patterns among AIS patients.

**Methods:**

A total of 219 eligible patients were enrolled at the stroke centers of two tertiary hospitals in Xuzhou, China from April 2023 to June 2024. The Center for Epidemiologic Studies Depression Scale (CES-D) was used to assess depressive symptoms during the acute hospitalization. The Group-based trajectory model was conducted to identify distinct trajectories of functional recovery, as measured by modified Rankin Scale (mRS) and Barthel Index (BI) at baseline, 3 months, and 6 months. A series of multinomial logistic regression models were performed to examine the relationship between early depressive symptoms and dynamic recovery patterns.

**Results:**

We identified 3 mRS trajectories (mild, moderate, and severe) and 5 BI trajectories (low-rapid rise, moderate low-stable, moderate-progressive rise, moderate high-rapid decline, and high-stable), respectively. After full adjustments, patients with early depressive symptoms were at increased likelihood of being in the moderate (OR 8.22, 95% CI 2.77–24.39) and severe (OR 24.41, 95% CI 5.33–111.90) trajectory group for mRS trajectories, and of the moderate high-rapid decline (OR 12.93, 95% CI 1.49–112.42) trajectory group for BI trajectories ( *p* < 0.05).

**Conclusion:**

Early depressive symptoms were associated with unfavorable functional recovery trajectories within 6 months following acute stroke in AIS patients.

## 1 Introduction

Depression is the most frequent psychiatric complication of stroke, characterized mainly by depressed mood and loss of interest (1). It was reported that approximately one-third of survivors would be affected by depression at any time of stroke up to 5 years (2). Depressive symptoms are very common in the acute stage of stroke among hospitalized patients, with prevalence ranging from 5 to 54% (3, 4). Patients with depression often have lower rehabilitation motivation, prolonged recovery duration, reduced quality of life, as well as higher mortality and stroke recurrence compared to those with non-depressed stroke (1, 5), which may be ascribed to biological changes of neuroplasticity resulting from stroke-induced insults, and/or psychosocial changes (6). Although the presence of depression indicates poor stroke outcomes, it is frequently overlooked or inadequately managed in clinical practice (7). Only 6% of acute stroke patients and 32% of recovery-phase patients received specialized psychological support (8). Therefore, the early detection and management of depression during the acute phase in stroke patients may be key to preventing subsequent adverse clinical outcomes.

Functional deficit is an expected consequence of stroke, and patients often experience functional disability (e.g., hemiplegia, aphasia, cognitive impairment, etc) and decreased ability in activities of daily living (ADL). The process of recovery after stroke demonstrates dynamic changes with great population heterogeneity (9, 10). However, to date, most studies have evaluated functional status at a specific time point, failing to reflect the dynamic nature of recovery (11–13). Several longitudinal studies employed traditional models (e.g., linear mixed model) to account for variations in functional recovery and time trends among individuals (14–16); however, these models may be limited in identifying latent heterogeneity in trajectories within certain groups of individuals (17). Despite limited evidence suggesting that early depression or depressive symptoms are associated with negative functional outcomes (18–20), few studies have examined the impact of acute-phase depressive symptoms on heterogeneous trajectories of functional recovery in patients with acute ischemic stroke (AIS).

In this contex, the Group-based trajectory modeling (GBTM) may offer a promising solution, as it is widely recognized for its effectiveness in depicting dynamic change and identifying potential heterogeneity in trajectories among the study population (17, 21). Accordingly, we aimed to apply GBTM for identifying distinct trajectories in patients following similar evolution of functional recovery during the first 6 months after stroke onset. Based on the classification of identified trajectories for functional recovery, we further investigated the relationship between early depressive symptoms and these recovery patterns over time in AIS patients.

## 2 Materials and methods

### 2.1 Study design and patients

This study was a longitudinal study conducted at the stroke centers of two tertiary hospitals from April 2023 to June 2024 in Xuzhou, China. The inclusion criteria for patients were as follows: (1) diagnosed with AIS by a neurologist according to a focal neurologic deficit and a corresponding infarct on computed tomography (CT) or magnetic resonance imaging (MRI), (2) age 18 years or older, and (3) <2 weeks since stroke onset. The exclusion criteria were as follows: (1) diagnosed with mental disorder, cognitive or emotional impairment prior to stroke onset, (2) having a transient ischemic attack (TIA), and (3) life expectancy < 6 months.

### 2.2 Procedures

Two trained neurologists recruited eligible patients for this study. Two trained research assistants interviewed patients and their primary family caregivers in person to collect sociodemographic and stroke-related information, combined with medical records.

Baseline assessment (T1) was performed during the acute hospitalization of patients (within 7–10 days following acute stroke) and follow-up assessments were conducted by telephone interviews at 3 months (T2) and 6 months (T3) after stroke onset (22, 23). Supplementary Table S1 presents the measures collected at each time point.

### 2.3 Measures

#### 2.3.1 Assessment of depressive symptoms

Depressive symptoms were measured using the Center for Epidemiologic Studies Depression Scale (CES-D) (24), which reflected the frequency of depressive symptoms in participants during the past week. CES-D consists of 20 items, including 4 domains: depressed affect, positive affect, somatic and retarded activity, and interpersonal problems. Each item of CES-D ranged from 0 (little or none at all [<1 day]) to 3 [most or all of the time (5–7 days)] on a 4-point Likert scale, with four items scored in reverse due to positive statements. The total scores ranged from 0 to 60, with higher scores indicating higher level of depressive symptoms, and stroke patients with the score of 16 or more were considered to have depressive symptoms (25). CES-D has been widely used for depression screening in Chinese population (26, 27) and has been proved to have good reliability and validity among stroke patients (25, 28).

#### 2.3.2 Assessment of functional outcomes

The modified Rankin Scale (mRS), as a reliable instrument for evaluating functional status, was widely used to assess the degree of dependence or disability in stroke survivors (29). The scale scores ranged from 0 (no symptoms at all) to 6 (death), with higher scores indicating more severe disability or dependence (29). The mRS score of 0–1 was usually categorized as a favorable functional outcome in clinical research or practice, while mRS score of 2–5 represented an unfavorable outcome (30, 31).

The Barthel index (BI) was used to measure patients’ ability to perform 10 basic ADLs (32). The scale scores ranged from 0 to 100, including self-care (feeding, grooming, bathing, dressing, bowel and bladder care, and toilet use) and mobility (ambulation, transfers, and stair climbing), and higher scores indicated greater independence (32, 33). The BI was the most commonly used ADL measurement that provided information for stroke patients to develop long-term care and rehabilitation plans (34).

### 2.4 Covariates

The pertinent covariates collected at baseline (T1) were composed of sociodemographic factors (age, sex, marital status, educational level, residence, occupational status, household income/yuan per year), severity of stroke [National Institutes of Health Stroke Scale (NIHSS)] (35, 36), classification of stroke [Oxfordshire Community Stroke Project (OCSP) classification, Trial of ORG 10172 in Acute Stroke Treatment (TOAST) classification] (37, 38), therapeutic options (intravenous thrombolysis (39, 40), endovascular thrombectomy (41, 42), conservative therapy (39, 43)), medical comorbidities (hypertension, diabetes, dyslipidemia, atrial fibrillation), risk factors (history of stroke, smoking status, drinking status, body mass index [BMI]), and stroke rehabilitation. A detailed coding of covariates is listed in Supplementary Table S2. Endovascular treatment included intra-arterial thrombectomy, arterial thrombolysis, and emergency angioplasty within 6 to 24 h of stroke onset (44). BMI was calculated by weight (kg) divided by square of height (m^2^), and ≥24 kg/m^2^ was defined as overweight or obesity referring to the Chinese criteria for adults (45). Stroke rehabilitation was provided by occupational therapy, physical therapy, and speech and language therapy during hospitalization (46).

### 2.5 Data quality assurance

To ensure data quality, all neurologists and research assistants involved in data collection received standardized and homogeneous training, which covered interpretation of the study protocol, standardized operation procedures for scales, interview skills, and data recording requirements. Only those who passed the post-training assessment were permitted to participate in the study, so as to ensure the consistency of assessments. A database was established using structured electronic data capture forms with EpiData 3.1 software, where logic checks and range restrictions were set to prevent invalid data entry. All data underwent double data entry by two independent research assistants, followed by cross-verification to ensure the accuracy of data entry.

### 2.6 Statistical analyses

The GBTM assumes that the population has inter-individual heterogeneity and aims to identify homogeneous classes of individuals following similar developmental trajectories over time (17). We applied GBTM to identify potential trajectories of mRS and BI from stroke onset to recovery period (within 6 months) in AIS patients (47). The trajectory models tested from 2 trajectory classes to 6 trajectory classes (i.e., linear, quadratic, and cubic) were fitted until the best model was determined. The optimal number of mRS or BI trajectory groups was determined based on the following criteria: (1) Bayesian information criterion (BIC), with a lower absolute value indicating a better fit; (2) average posterior probability (AvePP) of assigning an individual to a trajectory group ≥ 0.70; and (3) proportion of the participants belonging to each trajectory group > 5% (21, 48). Trajectories of mRS or BI were analyzed with the lcmm package (49).

Baseline characteristics of AIS patients were described as numbers (n) and proportion (%) for categorical variables, while continuous variables were described as mean ± standard deviation (SD) or median and interquartile range (IQR). Analysis of variance, Kruskal-Wallis H test, Chi-square test, or Fisher’s exact test were conducted to compare the baseline characteristics of included AIS patients across distinct trajectory groups. A series of multinomial logistic regression models were performed to explore the association between early depressive symptoms and trajectories of functional recovery (mRS, BI) over time. Firstly, we ran the univariate analysis to test the association between early depressive symptoms, covariates and functional recovery trajectories, respectively (Supplementary Tables S3, S4). Then, only those significant ( *p*<0.05) univariate factors were allowed to enter into the final multivariate logistic regression models, and multicollinearity was assessed among all variables using the variance inflation factor. To assess the effect of early depressive symptoms on trajectories of functional recovery, we incorporated covariates adjustment into the models in turn. For the mRS trajectories, (1) Model I was the base model adjusting for baseline depressive symptoms; (2) Model II: model I plus stroke-related factors (NIHSS, stroke rehabilitation, OCSP classification, hypertension, diabetes, atrial fibrillation, and smoking status); (3) Model III: model II plus sociodemographic factors (age, sex, and marital status). For the BI trajectories, (1) Model I was the base model adjusting for baseline depressive symptoms; (2) Model II: model I plus stroke-related factors (NIHSS, stroke rehabilitation, OCSP classification, hypertension, and diabetes); (3) Model III: model II plus sociodemographic factor (sex). Odds ratios (ORs) and 95% confidence intervals (CIs) for the effect of early depressive symptoms on mRS or BI trajectories were reported.

To further assess the robustness of the set of logistic regression models, the E-values were computed to evaluate the minimum strength of association, on the OR scale, that an unmeasured confounder would need to have with both early depressive symptoms and distinct trajectories of functional recovery to fully nullify the observed association, conditional on the measured covariates (50). Considering the sample size distribution among BI trajectory subgroups and the stability of the model, another sensitivity analysis was performed by excluding the small “moderate high–rapid decline” subgroup (accounting for 8.3% of the sample). The BI trajectory model was refitted to re-examine the association between early depressive symptoms and functional recovery trajectories. All statistical analyses were performed using R software version 4.3.2 and IBM SPSS version 26.0. The statistical significance was set at two-sided *p* < 0.05 throughout.

## 3 Results

### 3.1 Sample characteristics

**Figure 1:**
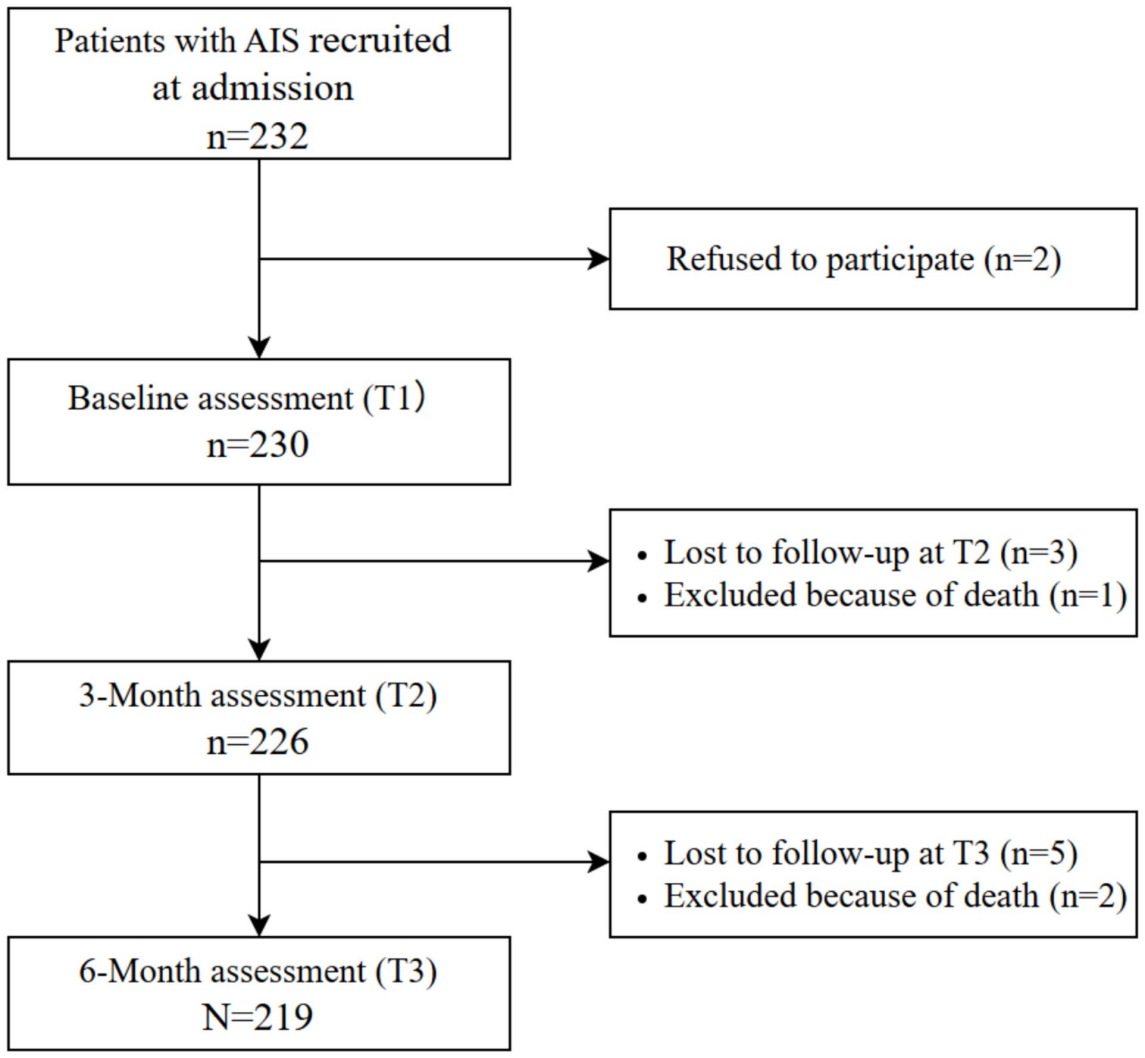
Flow diagram of patients with acute ischemic stroke (AIS).

Figure 1 shows the flow diagram of AIS patients assessed at each time point. In this study, we included patients who had mRS and BI measurements at three time points (T1, T2, and T3) and excluded those with missing data due to loss to follow-up or death. A total of 219 patients were enrolled in the study. Participants who withdrew from the study (*n* = 11) showed no significant differences in depressive symptoms ( *p* = 0.217) at baseline, age ( *p* = 0.468), gender ( *p* = 0.154), marital status ( *p* = 0.067), and comorbidities ( *p* > 0.05), but had higher NIHSS scores ( *p* = 0.007). It could be explained that those patients were more likely to discontinue their participation from the study due to more severe brain damage. Table 1 presents the baseline characteristics of the study patients, which was consistent with those of stroke patients reported by the China National Stroke Registry (CNSR) (51, 52).

**Table 1:** Baseline characteristics of patients with acute ischemic stroke (*N* = 219).

Characteristics	Value
Age (years), mean ±SD	66.7 ± 9.90
Sex, *n* (%)	
Male	143 (65.3)
Female	76 (34.7)
Marital status, *n* (%)	
Married	180 (82.2)
Divorced/single/widowed	39 (17.8)
Educational level, *n* (%)	
Illiterate	21 (9.6)
Primary school	31 (14.2)
Middle school	60 (27.4)
High school	64 (29.2)
University/college or above	43 (19.6)
Residence, *n* (%)	
Urban	191 (87.2)
Rural	28 (12.8)
Occupational status, *n* (%)	
Employed	31 (14.2)
Retired	163 (74.4)
Unemployed	25 (11.4)
Household income/yuan per year, *n* (%)	
Under 50,000 yuan	64 (29.2)
Among 50,000–100,000 yuan	114 (52.1)
Over 100,000 yuan	41 (18.7)
NIHSS, median (IQR)	7 (3–11)
OCSP classification, *n* (%)	
TACI	22 (10.0)
PACI	57 (26.0)
POCI	63 (28.8)
LACI	77 (35.2)
TOAST classification, *n* (%)	
LAA	70 (32.0)
CE	32 (14.6)
SAO	74 (33.8)
ODE	39 (17.8)
UDE	4 (1.8)
Therapeutic options, *n* (%)	
Intravenous thrombolysis	26 (11.9)
Intra-arterial thrombectomy	4 (1.8)
Conservative therapy	189 (86.3)
Comorbidities, *n* (%)	
Hypertension	166 (75.8)
Diabetes	95 (43.4)
Dyslipidemia	44 (20.1)
Atrial fibrillation	32 (14.6)
Risk factors	
History of stroke, *n* (%)	47 (21.4)
Smoking status, *n* (%)	
Former smoker	44 (20.1)
Never smoker	126 (57.5)
Current smoker	49 (22.4)
Drinking status, *n* (%)	
Former drinker	23 (10.5)
Never drinker	122 (55.7)
Current drinker	74 (33.8)
BMI (≥24 kg/m^2^), *n* (%)	116 (53.0)
Stroke rehabilitation, *n* (%)	57 (26.0)
Depressive symptoms, *n* (%)	118 (53.9)

NIHSS, National Institutes of Health Stroke Scale; OCSP, Oxfordshire Community Stroke Project; TACI, total anterior circulation infarct; PACI, partial anterior circulation infarct; POCI, posterior circulation infarct; LACI, lacunar circulation infarcts; TOAST, Trial of ORG 10172 in Acute Stroke Treatment; LAA, Large-artery atherothrombotic; CE, Cardioembolic; SAO, Small-artery occlusion; ODE, Other determined etiology; UDE, Undetermined etiology; BMI, body mass index; *n*, number; SD, Standard Deviation; IQR, Interquartile.

### 3.2 Trajectories of mRS and baseline characteristics

**Figure 2:**
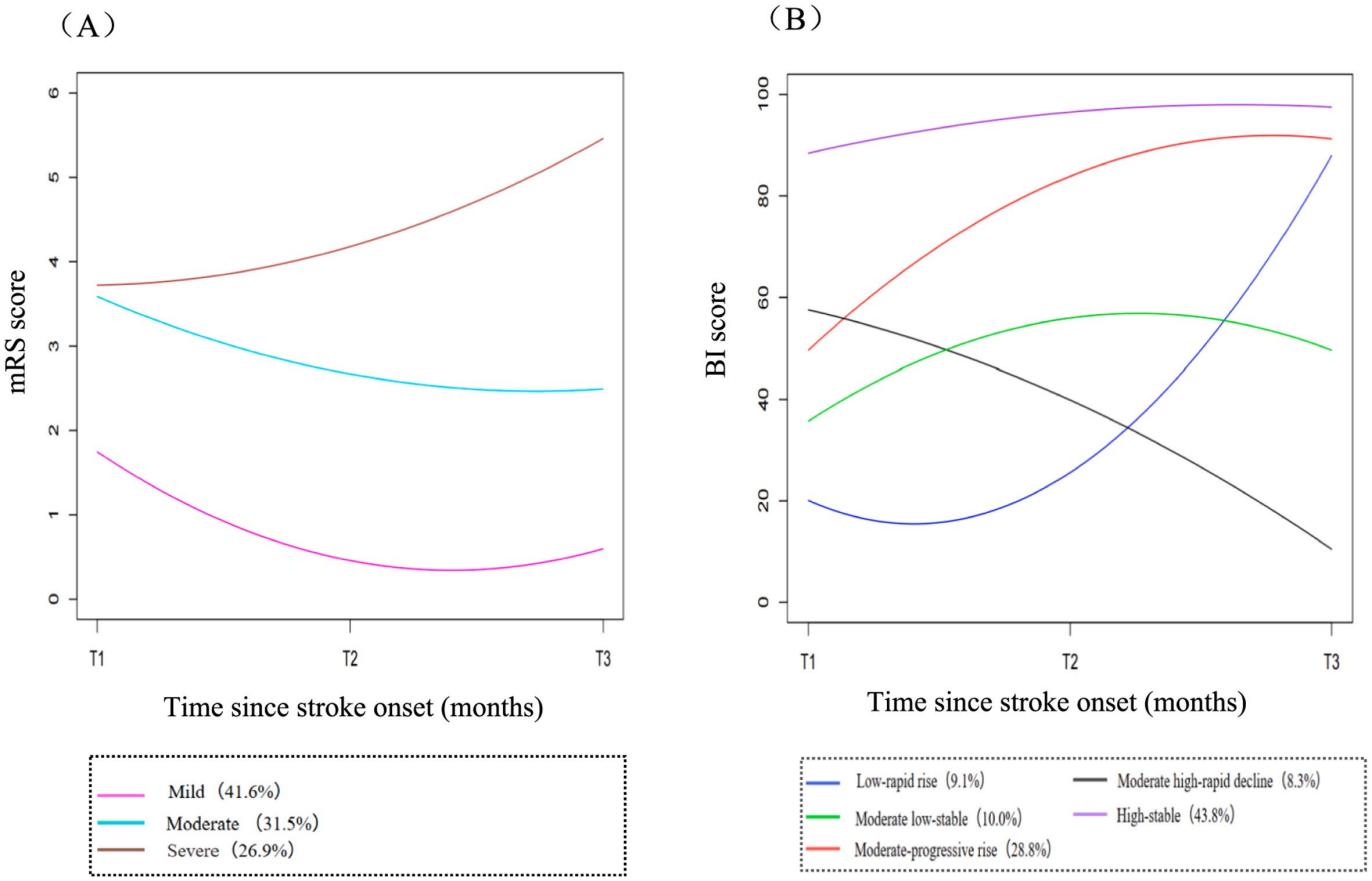
Trajectories of functional recovery. **(A)** shows the identified trajectories of mRS score across different severity groups. **(B)** presents the trajectories of BI score across different recovery patterns. mRS, modified Rankin Scale; BI, Barthel Index. T1, baseline; T2, 3months; T3, 6 months; T1 to T3 represent the assessments performed at different time points.

Three distinct mRS trajectory groups were identified as the best model according to the recommended statistical criteria (Figure 2A; Supplementary Table S5). The AIS patients were divided into 3 groups based on their mRS trajectories: Group 1 (mild trajectory, 41.6%) contained 91 patients with mRS scores below 2 during the first 6 months after stroke onset, indicating excellent functional recovery; Group 2 (moderate trajectory, 31.5%) contained 69 patients with mRS scores began at close to 4 during acute hospitalization and gradually decreased to around 3 during 3-month follow-up and then maintained this level until 6-month follow-up, indicating poor functional recovery; Group 3 (severe trajectory, 26.9%) contained 59 patients with mRS scores began at around 4 but then continued to increase rapidly until the end of the follow-up, indicating worsening functional recovery. Longitudinal changes in functional status after stroke onset across different mRS trajectory groups are shown in Supplementary Figure S1.

Table 2 presents the sociodemographic and clinical characteristics of patients in three mRS trajectory groups. Patients in the severe trajectory were more likely to be older and unmarried, had a greater proportion of posterior circulation infarct (POCI), and higher admission NIHSS score, whereas patients in the mild trajectory were more likely to be women, had a greater proportion of lacunar circulation infarcts (LACI) and small artery occlusion (SAO), and a lower burden of hypertension, diabetes, atrial fibrillation and stroke rehabilitation ( *p* < 0.05).

**Table 2:** Characteristics of patients by mRS trajectory groups (*n* = 219).

Characteristics	Trajectories of mRS			*p* value
	Mild	Moderate	Severe	
	(*n* = 91)	(*n* = 69)	(*n* = 59)	
Age (years), mean ±SD	65.3 ± 9.8	65.3 ± 9.2	70.4 ± 9.8	**0.003**
Sex, *n* (%)				**0.025**
Male	50 (54.9)	50 (72.5)	43 (72.9)	
Female	41 (45.1)	19 (27.5)	16 (27.1)	
Marital status, *n* (%)				**0.005**
Married	82 (90.1)	57 (82.6)	41 (69.5)	
Divorced/single/widowed	9 (9.9)	12 (17.4)	18 (30.5)	
Educational level, *n* (%)				0.944
Illiterate	9 (9.9)	7 (10.1)	5 (8.5)	
Primary school	11 (12.1)	12 (17.4)	8 (13.6)	
Middle school	23 (25.3)	17 (24.6)	20 (33.9)	
High school	29 (31.9)	19 (27.5)	16 (27.1)	
University/college or above	19 (20.9)	14 (20.3)	10 (16.9)	
Residence, *n* (%)				0.360
Urban	82 (90.1)	57 (82.6)	52 (88.1)	
Rural	9 (9.9)	12 (17.4)	7 (11.9)	
Occupational status, *n* (%)				0.586
Employed	15 (16.5)	9 (13.0)	7 (11.9)	
Retired	67 (73.6)	49 (71.0)	47 (79.7)	
Unemployed	9 (9.9)	11 (16.0)	5 (8.5)	
Household income/yuan per year, *n* (%)				0.101
Under 50,000 yuan	19 (20.9)	23 (33.3)	22 (37.3)	
Among 50,000–100,000 yuan	57 (62.6)	32 (46.4)	25 (42.4)	
Over 100,000 yuan	15 (16.5)	14 (20.3)	12 (20.3)	
NIHSS, median (IQR)	3 (2–4)	10 (7–12)	11 (8–14)	**<0.001**
OCSP classification, *n* (%)				**0.007**
TACI	7 (7.7)	10 (14.5)	5 (8.5)	
PACI	20 (22.0)	19 (27.5)	18 (30.5)	
POCI	19 (20.9)	20 (29.0)	24 (40.7)	
LACI	45 (49.5)	20 (29.0)	12 (20.3)	
TOAST classification, *n* (%)				**0.002**
LAA	17 (18.7)	30 (43.5)	23 (39.0)	
CE	16 (17.6)	7 (10.1)	9 (15.3)	
SAO	45 (49.5)	15 (21.7)	14 (23.7)	
ODE	12 (13.2)	16 (23.2)	11 (18.6)	
UDE	1 (1.1)	1 (1.4)	2 (3.4)	
Therapeutic options, *n* (%)				0.136
Intravenous thrombolysis	13 (14.3)	9 (13.0)	4 (6.8)	
Intra-arterial thrombectomy	0	1 (1.5)	3 (5.1)	
Conservative therapy	78 (85.7)	59 (85.5)	52 (88.1)	
Comorbidities, *n* (%)				
Hypertension	61 (67.0)	57 (82.6)	48 (81.4)	**0.038**
Diabetes	28 (30.8)	37 (53.6)	30 (50.8)	**0.006**
Dyslipidemia	16 (17.6)	11 (16.0)	17 (28.8)	0.143
Atrial fibrillation	8 (8.8)	16 (23.2)	8 (13.6)	**0.037**
BMI (≥24 kg/m^2^), *n* (%)	55 (60.4)	34 (49.3)	27 (45.8)	0.162
History of stroke, *n* (%)	16 (17.6)	16 (23.2)	15 (25.4)	0.476
Smoking status, *n* (%)				0.124
Current smoker	23 (25.3)	19 (27.5)	7 (11.9)	
Never smoker	52 (57.1)	39 (56.5)	35 (59.3)	
Former smoker	16 (17.6)	11 (16.0)	17 (28.8)	
Drinking status, *n* (%)				0.122
Current drinker	27 (29.7)	31 (44.9)	16 (27.1)	
Never drinker	54 (59.3)	34 (49.3)	34 (57.6)	
Former drinker	10 (11.0)	4 (5.8)	9 (15.3)	
Stroke rehabilitation, *n* (%)	8 (8.8)	29 (42.0)	20 (33.9)	**<0.001**

mRS, modified Rankin Scale; NIHSS, National Institutes of Health Stroke Scale; OCSP, Oxfordshire Community Stroke Project; TACI, total anterior circulation infarct; PACI, partial anterior circulation infarct; POCI, posterior circulation infarct; LACI, lacunar circulation infarcts; TOAST, Trial of ORG 10172 in Acute Stroke Treatment; LAA, Large-artery atherothrombotic; CE, Cardioembolic; SAO, Small-artery occlusion; ODE, Other determined etiology; UDE, Undetermined etiology; BMI, body mass index; n, number; SD, Standard Deviation; IQR, Interquartile. Values in bold indicate statistical significance with ( *p* < 0.05).

### 3.3 Trajectories of BI and baseline characteristics

Five distinct BI trajectory groups were similarly identified by our model based on the criteria and clinical interpretability (Figure 2B; Supplementary Table S5): Group1(low-rapid rise trajectory, 9.1%) included 20 patients whose BI scores were the lowest in the acute stage then rose rapidly over time, representing a gradual improvement in ADL; Group 2 (moderate low-stable trajectory, 10.0%) included 22 patients with BI scores started below 40 then stabilized between 50 and 55; Group 3 (moderate-progressive rise trajectory, 28.8%) included 63 individuals and followed a similar pattern to the group 2, with BI scores fluctuating between 50 and 90, and these two groups exhibited mild to moderate dependence in ADL; Group 4 (moderate high-rapid decline trajectory, 8.3%) included 18 patients referring to those who initially had BI levels similar to the group 3 but continued to decline acutely over the following time, representing a progressive deterioration in ADL; Group 5 (high-stable trajectory, 43.8%) included 96 patients with persistently high BI levels during the first 6 months after onset, representing the functional independence. The functional changes following acute stroke between different BI trajectory groups are shown in Supplementary Figure S2.

The sociodemographic and clinical profiles of patients by BI trajectories are shown in Table 3. Patients in the high-stable trajectory tended to have lower NIHSS scores on admission, a higher proportion of SAO, and a lower rate of stroke rehabilitation, whereas those in the low-rapid rise trajectory were more likely to carry a greater burden of hypertension and diabetes ( *p* < 0.05).

**Table 3:** Characteristics of patients by BI trajectory groups (*n* = 219).

Characteristics	Trajectories of BI					*p* value
	Low-rapid rise	Moderate low-stable	Moderate-progressive rise	Moderate high-rapid decline	High-stable	
	(*n* = 20)	(*n* = 22)	(*n* = 63)	(*n* = 18)	(*n* = 96)	
Age (years), mean ±SD	68.2 ± 11.2	67.4 ± 8.3	67.6 ± 9.2	69.8 ± 11.1	65.0 ± 10.1	0.210
Sex, *n* (%)						0.171
Male	17 (85.0)	16 (72.7)	41 (65.1)	13 (72.2)	56 (58.3)	
Female	3 (15.0)	6 (27.3)	22 (34.9)	5 (27.8)	40 (41.7)	
Marital status, *n* (%)						0.300
Married	15 (75.0)	19 (86.7)	49 (77.8)	13 (72.2)	84 (87.5)	
Divorced/single/widowed	5 (25.0)	3 (13.6)	14 (22.2)	5 (27.8)	12 (12.5)	
Educational level, *n* (%)						0.414
Illiterate	1 (5.0)	4 (18.2)	7 (11.1)	1 (5.6)	8 (8.3)	
Primary school	2 (10.0)	1 (4.5)	11 (17.5)	4 (22.2)	13 (13.5)	
Middle school	10 (50.0)	4 (18.2)	12 (19.0)	7 (38.9)	27 (28.1)	
High school	3 (15.0)	8 (36.4)	18 (28.6)	4 (22.2)	31 (32.3)	
University/college or above	4 (20.0)	5 (22.7)	15 (23.8)	2 (11.1)	17 (17.7)	
Residence, *n* (%)						0.535
Urban	18 (90.0)	17 (77.3)	56 (88.9)	17 (94.4)	83 (86.5)	
Rural	2 (10.0)	5 (22.7)	7 (11.1)	1 (5.6)	13 (13.5)	
Occupational status, *n* (%)						0.871
Employed	3 (15.0)	3 (13.6)	7 (11.1)	2 (11.1)	16 (16.7)	
Retired	16 (80.0)	16 (72.7)	46 (73.0)	15 (83.3)	70 (72.9)	
Unemployed	1 (5.0)	3 (13.6)	10 (15.9)	1 (5.6)	10 (10.4)	
Household income/yuan per year, *n* (%)						0.197
Under 50,000 yuan	9 (45.0)	6 (27.3)	17 (27.0)	9 (50.0)	23 (24.0)	
Among 50,000–100,000 yuan	7 (35.0)	10 (45.5)	32 (50.8)	8 (44.4)	57 (59.4)	
Over 100,000 yuan	4 (20.0)	6 (27.3)	14 (22.2)	1 (5.6)	16 (16.7)	
NIHSS, median (IQR)	15 (9.3–17)	13 (10–17)	10 (7–12)	9.5 (7–11.3)	3 (2–5)	**<0.001**
OCSP classification, *n* (%)						0.066
TACI	4 (20.0)	3 (13.6)	7 (11.1)	2 (11.1)	6 (6.3)	
PACI	4 (20.0)	9 (40.9)	15 (23.8)	6 (33.3)	23 (24.0)	
POCI	8 (40.0)	4 (18.2)	24 (38.1)	6 (33.3)	21 (21.9)	
LACI	4 (20.0)	6 (27.3)	17 (27.0)	4 (22.2)	46 (47.9)	
TOAST classification, *n* (%)						**0.005**
LAA	8 (40.0)	13 (59.1)	19 (30.2)	7 (38.9)	23 (24.0)	
CE	4 (20.0)	2 (9.1)	8 (12.7)	5 (27.8)	13 (13.5)	
SAO	3 (15.0)	5 (22.7)	17 (27.0)	3 (16.7)	46 (47.9)	
ODE	4 (20.0)	1 (4.5)	19 (30.2)	3 (16.7)	12 (12.5)	
UDE	1 (5.0)	1 (4.5)	0	0	2 (2.1)	
Therapeutic options, *n* (%)						0.086
Intravenous thrombolysis	1 (5.0)	3 (13.6)	9 (14.3)	0	13 (13.5)	
Intra-arterial thrombectomy	0	2 (9.1)	2 (3.2)	0	0	
Conservative therapy	19 (95.0)	17 (77.3)	52 (82.5)	18 (100.0)	83 (86.5)	
Comorbidities, *n* (%)						
Hypertension	18 (90.0)	19 (86.4)	52 (82.5)	14 (77.8)	63 (65.6)	**0.032**
Diabetes	14 (70.0)	9 (40.9)	34 (54.0)	8 (44.4)	30 (31.3)	**0.006**
Dyslipidemia	7 (35.0)	5 (22.7)	11 (17.5)	6 (33.3)	15 (15.6)	0.178
Atrial fibrillation	4 (20.0)	5 (22.7)	10 (15.9)	3 (16.7)	10 (10.4)	0.538
BMI (≥24 kg/m^2^), *n* (%)	11 (55.0)	9 (40.9)	37 (58.7)	6 (33.3)	53 (55.2)	0.274
History of stroke, *n* (%)	4 (20.0)	5 (22.7)	18 (28.6)	4 (22.2)	16 (16.7)	0.517
Smoking status, *n* (%)						0.247
Current smoker	4 (20.0)	4 (18.2)	15 (23.8)	0	26 (27.1)	
Never smoker	11 (55.0)	11 (50.0)	37 (58.7)	12 (66.7)	55 (57.3)	
Former smoker	5 (25.0)	7 (31.8)	11 (17.5)	6 (33.3)	15 (15.6)	
Drinking status, *n* (%)						0.429
Current drinker	10 (50.0)	9 (40.9)	22 (34.9)	2 (11.1)	31 (32.3)	
Never drinker	8 (40.0)	10 (45.5)	35 (55.6)	13 (72.2)	56 (58.3)	
Former drinker	2 (10.0)	3 (13.6)	6 (9.5)	3 (16.7)	9 (9.4)	
Stroke rehabilitation, *n* (%)	10 (50.0)	13 (59.1)	20 (31.7)	6 (33.3)	8 (8.3)	**<0.001**

BI, Barthel Index; NIHSS, National Institutes of Health Stroke Scale; OCSP, Oxfordshire Community Stroke Project; TACI, total anterior circulation infarct; PACI, partial anterior circulation infarct; POCI, posterior circulation infarct; LACI, lacunar circulation infarcts; TOAST, Trial of ORG 10172 in Acute Stroke Treatment; LAA, Large-artery atherothrombotic; CE, Cardioembolic; SAO, Small-artery occlusion; ODE, Other determined etiology; UDE, Undetermined etiology; BMI, body mass index; n, number; SD: Standard Deviation; IQR, Interquartile. Values in bold indicate statistical significance with ( *p* < 0.05).

### 3.4 Early depressive symptoms and trajectories of functional recovery

Tables 4, 5 present the association between early depressive symptoms and trajectories of functional recovery (mRS, BI) during the 6-month follow-up period after onset of stroke in multivariable logistic regression models with adjustment for relevant covariates, respectively.

**Table 4:** Association between depressive symptoms and trajectories of mRS.

Variables	Moderate vs. Mild		Severe vs. Mild	
	OR (95% CI)	*p* value	OR (95% CI)	*p* value
Model I				
Depressive symptoms	12.54 (5.88, 26.77)	**<0.001**	53.25 (14.96, 189.55)	**<0.001**
Model II				
Depressive symptoms	8.47 (2.96, 24.20)	**<0.001**	30.21 (6.83, 133.57)	**<0.001**
NIHSS	1.61 (1.33, 1.95)	**<0.001**	1.72 (1.40, 2.10)	**<0.001**
Stroke rehabilitation	1.58 (0.38, 6.60)	0.531	1.08 (0.23, 5.01)	0.921
OCSP classification				
TACI	0.46 (0.08, 2.71)	0.388	0.66 (0.09, 5.13)	0.694
PACI	1.05 (0.30, 3.71)	0.940	1.57 (0.37, 6.77)	0.543
POCI	1.29 (0.36, 4.64)	0.695	2.44 (0.55, 10.90)	0.243
LACI (ref)				
Hypertension	1.15 (0.35, 3.81)	0.818	1.10 (0.27, 4.52)	0.897
Diabetes	2.38 (0.83, 6.83)	0.106	1.91 (0.57, 6.37)	0.293
Atrial fibrillation	0.85 (0.18, 3.99)	0.833	0.35 (0.06, 2.05)	0.243
Smoking status				
Former smoker	0.26 (0.05, 1.26)	0.095	1.32 (0.20, 8.65)	0.776
Never smoker	0.61 (0.18, 2.07)	0.427	2.59 (0.53, 12.62)	0.240
Current smoker (ref)				
Model III				
Depressive symptoms	8.22 (2.77, 24.39)	**<0.001**	24.41 (5.33, 111.90)	**<0.001**
NIHSS	1.62 (1.34, 1.96)	**<0.001**	1.70 (1.38, 2.09)	**<0.001**
Stroke rehabilitation	1.71 (0.40, 7.28)	0.466	1.39 (0.29, 6.81)	0.682
OCSP classification				
TACI	0.37 (0.06, 2.41)	0.296	0.76 (0.09, 6.54)	0.802
PACI	1.00 (0.25, 3.97)	0.998	2.22 (0.43, 11.43)	0.339
POCI	1.04 (0.28, 3.86)	0.957	2.35 (0.49, 11.28)	0.287
LACI (ref)				
Hypertension	1.40 (0.39, 5.02)	0.602	1.30 (0.27, 6.28)	0.744
Diabetes	2.43 (0.81, 7.30)	0.114	1.73 (0.49, 6.05)	0.391
Atrial fibrillation	0.70 (0.14, 3.56)	0.664	0.23 (0.04, 1.48)	0.121
Smoking status				
Former smoker	0.27 (0.05, 1.38)	0.115	1.21 (0.17, 8.76)	0.851
Never smoker	1.83 (0.40, 8.36)	0.434	4.82 (0.71, 32.76)	0.108
Current smoker (ref)				
Age	0.99 (0.93, 1.04)	0.605	1.05 (0.98, 1.12)	0.198
Sex (Male)	4.61 (0.97, 19.67)	0.059	4.45 (0.88, 22.46)	0.071
Marital status (Married)	0.50 (0.11, 2.27)	0.372	0.25 (0.05, 1.31)	0.102

Model I: no adjustment; Model II: adjust for NIHSS, stroke rehabilitation, OCSP classification, hypertension, diabetes, atrial fibrillation, smoking status; Model III: NIHSS, stroke rehabilitation, OCSP classification, hypertension, diabetes, atrial fibrillation, smoking status, age, sex (male), marital status (married). mRS, modified Rankin Scale; NIHSS, National Institutes of Health Stroke Scale; OCSP, Oxfordshire Community Stroke Project; TACI, total anterior circulation infarct; PACI, partial anterior circulation infarct; POCI, posterior circulation infarct; LACI, lacunar circulation infarcts; OR: odds ratio, CI: confidence intervals; ref, Reference. Values in bold indicate statistical significance with ( *p* < 0.05).

**Table 5:** Association between depressive symptoms and trajectories of BI.

Variables	Low-rapid rise		Moderate low-stable		Moderate-progressive rise		Moderate high-rapid decline	
	vs High-stable		vs High-stable		vs High-stable		vs High-stable	
	OR (95% CI)	*p* value	OR (95% CI)	*p* value	OR (95% CI)	*p* value	OR (95% CI)	*p* value
Model I								
Depressive symptoms	38.00 (4.87, 296.71)	**0.001**	12.67 (3.49, 45.99)	**<0.001**	2.85 (1.48, 5.49)	**0.002**	34.00 (4.33,267.02)	**0.001**
Model II								
Depressive symptoms	8.28 (0.76, 90.19)	0.083	2.66 (0.49, 14.56)	0.261	0.94 (0.36, 2.460)	0.902	13.11 (1.51, 113.95)	**0.020**
NIHSS	2.57 (1.98, 3.34)	**<0.001**	2.47 (1.92, 3.18)	**<0.001**	1.81 (1.49, 2.19)	**<0.001**	1.69 (1.34, 2.12)	**<0.001**
Stroke rehabilitation	1.90 (0.36, 9.91)	0.449	2.77 (0.56, 13.61)	0.210	0.97 (0.28, 3.40)	0.964	1.06 (0.23, 4.98)	0.939
Hypertension	4.03 (0.51, 32.25)	0.188	4.76 (0.70, 32.41)	0.111	1.63 (0.54, 4.97)	0.390	1.43 (0.31, 6.59)	0.645
Diabetes	4.53 (0.99, 20.83)	0.052	1.12 (0.27, 4.62)	0.874	1.89 (0.73, 4.89)	0.187	1.17 (0.32, 4.25)	0.817
OCSP classification								
TACI	0.75 (0.06, 9.60)	0.828	0.36 (0.03, 4.29)	0.416	0.54 (0.08, 3.69)	0.532	0.90 (0.08, 10.49)	0.934
PACI	0.34 (0.04, 2.78)	0.311	0.81 (0.14, 4.73)	0.818	0.72 (0.21, 2.43)	0.593	1.01 (0.19, 5.34)	0.987
POCI	0.56 (0.09, 3.71)	0.550	0.25 (0.04, 1.64)	0.147	0.91 (0.29, 2.84)	0.872	0.98 (0.19, 5.16)	0.985
LACI (ref)								
Model III								
Depressive symptoms	8.55 (0.75, 97.47)	0.084	2.59 (0.47, 14.36)	0.277	0.94 (0.36, 2.46)	0.897	12.93 (1.49, 112.42)	**0.021**
NIHSS	2.62 (2.00, 3.42)	**<0.001**	2.48 (1.92, 3.19)	**<0.001**	1.81 (1.49, 2.20)	**<0.001**	1.69 (1.34, 2.12)	**<0.001**
Stroke rehabilitation	1.68 (0.31, 8.99)	0.546	2.70 (0.55, 13.35)	0.223	0.99 (0.28, 3.50)	0.988	1.05 (0.22, 4.97)	0.950
Hypertension	5.72 (0.65, 50.13)	0.115	5.25 (0.73, 38.08)	0.101	1.58 (0.51, 4.85)	0.425	1.46 (0.31, 6.91)	0.637
Diabetes	5.04 (1.08, 23.62)	0.051	1.15 (0.28, 4.79)	0.848	1.88 (0.73, 4.85)	0.194	1.17 (0.32, 4.29)	0.813
OCSP classification								
TACI	0.79 (0.06, 9.89)	0.857	0.33 (0.03, 4.08)	0.391	0.54 (0.08, 3.69)	0.532	0.88 (0.08, 10.31)	0.918
PACI	0.34 (0.04, 2.98)	0.328	0.88 (0.15, 5.17)	0.887	0.73 (0.21,2.46)	0.606	1.03 (0.20, 5.42)	0.975
POCI	0.44 (0.06, 3.10)	0.407	0.25 (0.04, 1.73)	0.161	0.97 (0.30, 3.13)	0.957	1.00 (0.19, 5.39)	0.999
LACI (ref)								
Sex (Male)	3.44 (0.52, 22.92)	0.201	1.28 (0.27, 6.18)	0.758	0.81 (0.30, 2.22)	0.686	1.03 (0.25, 4.23)	0.965

Model I: no adjustment; Model II: adjust for NIHSS, Stroke rehabilitation, OCSP classification, hypertension, diabetes; Model III: NIHSS, Stroke rehabilitation, OCSP classification, hypertension, diabetes, sex (Male). BI, Barthel Index; NIHSS, National Institutes of Health Stroke Scale; OCSP, Oxfordshire Community Stroke Project; TACI, total anterior circulation infarct; PACI, partial anterior circulation infarct; POCI, posterior circulation infarct; LACI, lacunar circulation infarcts; OR: odds ratio, CI: confidence intervals; ref, Reference. Values in bold indicate statistical significance with ( *p*< 0.05).

For mRS trajectories (Table 4), the unadjusted model (Model I) revealed that compared with the mild trajectory group, early depressive symptoms increased odds of patients belonging to the moderate (OR 12.54, 95% CI 5.88–26.77) and severe (OR 53.25, 95% CI 14.96–189.55) trajectory group. After adjusting for sociodemographic (age, sex, and marital status) and stroke-related factors (NIHSS at admission, stroke rehabilitation, OCSP classification, hypertension, diabetes, atrial fibrillation, and smoking status), the fully adjusted model (Model III) confirmed the results. Additionally, stroke severity at baseline independently predicted poor recovery trajectories, either in unadjusted or adjusted analyses ( *p* < 0.001).

For BI trajectories (Table 5), the unadjusted model (Model I) demonstrated that early depressive symptoms were associated with an increased likelihood of patients being in the low-rapid rise (OR 38.0, 95% CI 4.87–296.71), moderate low-stable (OR 12.67, 95% CI 3.49–45.99), moderate-progressive rise (OR 2.85, 95% CI 1.48–5.49), and moderate high-rapid decline (OR 34.0, 95% CI 4.33–267.02) trajectory groups, compared to the high-stable trajectory group. After adjusting for all covariates (Model III), the association between early depressive symptoms and the first three trajectory groups was no longer significant, but remained significant for the moderate high-rapid decline trajectory group (OR 12.93, 95% CI 1.49–112.42). The stroke severity at baseline was also significantly related to the BI trajectory groups throughout models ( *p* < 0.001 ).

### 3.5 Sensitivity analyses

We calculated the E-values to assess the robustness of observed associations against potential unmeasured confounder factors, as shown in Supplementary Table S6. The E-values for the associations between early depressive symptoms and mRS trajectories were 5.18 (moderate vs. mild) and 9.35 (severe vs. mild). Similarly, for the relationship between early depressive symptoms and BI trajectories, the E-value for the OR was 6.65 in the comparison between moderate high-rapid decline and high-stable trajectory group. This indicates that the observed associations could potentially be explained by an unmeasured confounder factor associated with both the exposure and the outcome, with a relative risk greater than the reported E-values, conditional on the measured covariates. The refitted model identified three distinct BI recovery trajectories (Supplementary Figure S3): a “moderate-low functional recovery” (Class 1, 15.4%, BI slowly improved from ≈20 to ≈50), a “high-stability” (Class 2, 59.2%, BI ≈80 maintained from T1, set as reference), and a “gradual improvement”(Class 3, 25.4%, BI increased from ≈40 at T1 to ≈80 at T3). In the adjusted multinomial logistic regression (Supplementary Table S7), early depressive symptoms remained significantly associated with the less favorable “moderate-low functional recovery” trajectory (OR = 3.62, 95% CI: 1.36–9.67, *p* = 0.010) but not with the “gradual improvement” trajectory (OR = 4.70, 95% CI: 0.90–24.69, *p* = 0.067). This confirms that the core finding—linking early depressive symptoms specifically to unfavorable functional recovery trajectories—is robust and not substantially altered by the exclusion of the smallest subgroup.

## 4 Discussion

In this longitudinal study of patients with AIS, we identified distinct trajectory groups through the dynamic change patterns of functional recovery (mRS, BI) during the first 6 months after stroke onset. The three distinct mRS trajectories were characterized by mild, moderate, and severe, while five distinct BI trajectories were categorized as low-rapid rise, moderate low-stable, moderate-progressive rise, moderate high-rapid decline, and high-stable. We found that depressive symptoms in the acute stage of stroke were associated with worsening functional recovery trajectories within the 6-month poststroke period in AIS patients after adjustment for covariates. Specifically, patients with early depressive symptoms had the highest risk of mRS trajectories belonging to the severe trajectory group. In particular, patients with early depressive symptoms were uniquely associated with an increased probability of membership in the moderate high-rapid decline trajectory group compared to other BI trajectories. To our best knowledge, this is the first study to report the relationship between baseline depressive symptoms and the longitudinal trajectories of functional recovery in Chinese patients with AIS.

The functional recovery trajectory of post-stroke is heterogeneous among individuals and varies over time. Several studies have suggested that functional recovery after stroke continues to evolve in different patterns, such as improvement, deterioration, or stability in different subgroups (53, 54). Our finding of the five BI trajectories was in line with the result of a prior study (47), although there were some changes in the patterns of trajectories. Another study has reported four mRS trajectories including no significant, slight, severe to moderate, and persistent severe disability among ischemic stroke patients age≥22 years during one-year follow-up (55), whereas the no significant disability trajectory was not identified in our study. The main reason for the variation in the number of trajectories might be attributed to the older age of the patients in our study, who were therefore more likely to be in the severe trajectory group (26.9%). Furthermore, the differences could be partly explained by the sample size and the procedure of follow-up (e.g., assessment frequencies, evaluation methods, and observation duration).

Our study showed that depressive symptoms within hospitalization predicted poor functional outcomes following acute stroke, which was consistent with the findings of previous studies (3, 18, 19). Although the correlation between depressive symptoms and stroke-related outcomes has been confirmed using various measurements ranging from weeks or months to years after ischemic stroke (56, 57), few studies have investigated the impact of depressive symptoms in the acute stage on heterogeneous trajectories of functional recovery. Our study further supplemented previous research by identifying the dynamic patterns of functional recovery. Unlike single or two time-point assessments, evaluating the dynamic changes in functional recovery may provide deeper insights into the prognosis for AIS patients. Moreover, we found that in the fully adjusted models, patients with early depressive symptoms consistently had a higher likelihood of belonging to the “severe” mRS trajectory group or the “moderate high-rapid decline” BI trajectory group. The finding suggests that individuals who experience depressive symptoms in the acute stage of stroke would face greater challenges in rehabilitation. It highlights the importance of detecting depression in the early phase after stroke onset, and has significant clinical implications (58, 59). Stroke severity was also found to be significantly related to adverse recovery, underscoring the pivotal impact of initial neurological deficits on stroke outcomes. It is noteworthy that depressive symptoms in the acute stage is connected with biological changes, such as inflammation and impaired brain neuroplasticity, as well as behavioral changes, including reduced motivation in rehabilitation engagement, social participation, and lifestyle modifications, and these factors in turn have detrimental effects on functional outcomes after stroke (1).

Although depression after stroke is highly prevalent, and it’s a range of unfavorable stroke-related outcomes, only a minority of AIS patients receive early detection during hospitalization. Neurologists should therefore prioritize structured depression screening in the acute phase, as endorsed by the American Heart Association/American Stroke Association (AHA/ASA) (60). Our findings extend this generic recommendation by providing a data-driven framework for risk stratification and subsequent management. Specifically, early depressive symptoms serve not only as a marker of psychological distress but also as a robust indicator for identifying patients at the greatest risk of following the most adverse functional recovery trajectories, namely the “severe” mRS and “moderate high–rapid decline” BI pathways. Consequently, a positive screen in the acute phase should immediately categorize a patient into a high-risk surveillance group. For these individuals, standard post-discharge monitoring must be intensified, with the 3-month follow-up constituting a critical decision point to detect early functional decline. This trajectory-informed perspective enables clinicians to move beyond a one-size-fits-all approach, allowing for the timely escalation of rehabilitation intensity and the integration of mental health interventions precisely when they are most needed to alter the projected recovery course. Thus, by linking acute depressive symptomatology to probable long-term functional outcomes, our study argues for transforming depression screening from an isolated check-box into the initial step of a dynamic, personalized management protocol aimed at mitigating the risk of the poorest recoveries.

The current study made contributions to the early detection and effective management tailored to those patients at higher risk by firstly exploring the associations between acute-phase depressive symptoms and specific patterns of recovery over time based on the application of GBTM. The functional outcomes in this study were comprehensively assessed by two widely adopted and validated instruments from different domains, which helps clinicians capture subtle changes in functional status to formulate more detailed support strategies for stroke patients. However, several limitations of this study should be noted. First, as a symptom-rating scale rather than a diagnostic instrument, the CES-D may have overestimated the prevalence of depressive symptoms in the acute setting due to overlapping somatic items (e.g., fatigue, sleep disturbance) that could reflect neurological impairment rather than depressive affect. Although the CES-D had demonstrated reliability and validity in stroke populations (25, 28), this inherent measurement bias limited the specificity of the score during the early post-stroke period and could have led to an inflated estimation of symptom burden. Second, given that the median NIHSS score at admission was 7 (interquartile range, 2–11), and patients who dropped out during follow-up were older and had a severe stroke at baseline, which may result in the study population with predominantly moderate and mild stroke severity. The conclusions of this study should be interpreted and extrapolated cautiously to other AIS patients. Third, functional status was assessed at only three time points (T1-T3), and participants with missing mRS and/or BI measurements were excluded, whereas more ample data could provide a more accurate analysis of trajectory. Furthermore, while telephone interviews were employed to improve follow-up rates, particularly among patients with severe disability, this method may have compromised the accuracy of certain BI items (e.g., transfers and stair climbing) that require direct observation, potentially introducing measurement bias toward overestimating functional independence, despite evidence supporting the reliability of telephone-assessed mRS. (23) Fourth, only 8.3% of patients exhibited moderate high-rapid decline of function. We may have been underpowered to detect clinically significant differences duo to the uneven sample sizes between the groups of BI trajectories. However, it is recommended that the minimum sample size of each BI trajectory group is more than 5% of the total population (17). Fifth, while this longitudinal study adjusted for key covariates including stroke severity (NIHSS) and lesion characteristics (OCSP/TOAST classification), residual confounding due to unmeasured psychosocial factors (e.g., social support and coping styles) remained possible. Additionally, reverse causality could not be entirely eliminated, as depressive symptoms might themselves be influenced by post-stroke neurological deficits. Finally, this study depicted the recovery trajectories of AIS patients within the initial 6 months after acute stroke. However, the observed recovery trajectories may not fully represent long-term patterns of functional change, due to prolonged recovery periods in some patients (beyond 6 months or longer) (61), which may limit the conclusions of the study. Thus, studies with larger samples, more complete information on demographics, and extended follow-up are needed to further elucidate the longer-term relationship between depression and dynamic change of functional recovery.

## 5 Conclusion

In conclusion, three mRS trajectories and five BI trajectories were identified in patients with acute ischemic stroke. We found that depressive symptoms in the acute phase were associated with poorer trajectories of functional recovery within 6 months after stroke onset. Our findings further support the recommendations for early screening for and effective treatment of depression after stroke, as well as for targeted strategies to reduce functional disability, to broadly alleviate the enormous burden on stroke survivors worldwide.

## Data Availability

The original contributions presented in the study are included in the article/Supplementary material, further inquiries can be directed to the corresponding authors.
