# Supplementary material for: Association between early depressive symptoms after stroke and trajectories of functional recovery among patients with acute ischemic stroke: a longitudinal study
Source: Front Neurol. 2026 Jan 27;17:1737884. doi: 10.3389/fneur.2026.1737884 (PMC12888208; doi:10.3389/fneur.2026.1737884)
Supplement: Supplementary file 1 [file Supplementary_file_1.pdf]

Supplementary Material

1 Supplementary Tables

Table S1. Measures collected at each time point

	Measures	T1	T2	T3
Functional outcome	mRS	×	×	×
	BI	×	×	×
Depressive symptoms	CES-D	×		
Sociodemographic factors	Age	×		
	Sex	×		
	Marital status	×		
	Educational level	×		
	Residence	×		
	Occupational status	×		
	Household income	×		
Severity of stroke	NIHSS	×		
Classification of stroke	OCSP classification	×		
	TOAST classification	×		
Therapeutic options	Intravenous thrombolysis	×		
	Endovascular thrombectomy	×		
	Conservative therapy	×		
Comorbidities	Hypertension	×		
	Diabetes	×		
	Dyslipidemia	×		
	Atrial fibrillation	×		
Risk factors	History of stroke	×		
	Smoking status	×		
	Drinking status	×		
	BMI	×		

Stroke rehabilitation	×

Note：T1, Baseline assessment; T2, 3-Month assessment; T3, 6-Month assessment; Abbreviations: mRS, modified Rankin Scale; BI, Barthel Index; CES-D, center for epidemiologic studies depression scale; NIHSS, National Institutes of Health Stroke Scale; OCSP, Oxfordshire Community Stroke Project; TOAST, Trial of ORG 10172 in Acute Stroke Treatment; BMI, body mass index.

Table S2. The list of covariates coding

	Covariates	Coding	Continuous variable	Categorical variable
Sociodemographic factors	Age		×	
	Sex	Male=1, female=0		×
	Marital status	Married=1<br>Divorced/single/widowed=0		×
	Educational level	Illiterate=1<br>Primary school=2<br>Middle school=3<br>High school=4<br>University/college or above=5		×
	Residence	Urban=1, Rural=0		×
	Occupational status	Employed=1<br>Retired=2<br>Unemployed=3		×
	Household income/ yuan per year	Under 50,000 yuan=1<br>Among 50,000-100,000 yuan=2<br>Over 100,000 yuan=3		×
Severity of stroke	NIHSS		×	
Classification of stroke	OCSP classification	TACI=1, PACI=2, POCI=3, LACI=4		×
	TOAST classification	LAA=1, CE=2, SAO=3, ODE=4,UDE=5		×
Therapeutic options				×
	Intravenous thrombolysis<br>Endovascular thrombectomy<br>Conservative therapy	Intravenous thrombolysis=1<br>Endovascular thrombectomy=2<br>Conservative therapy=3		
Comorbidities	Hypertension	Yes=1, no=0		×
	Diabetes	Yes=1, no=0		×
	Dyslipidemia	Yes=1, no=0		×
	Atrial fibrillation	Yes=1, no=0		×
Risk factors	History of stroke	Yes=1, no=0		×

Smoking status	Current smoking=1<br>Never smoking=2<br>Former smoking=3	×
Drinking status	Current drinking=1<br>Never drinking=2<br>Former drinking=3	×
BMI		×
Stroke rehabilitation	Yes=1, no=0	×

Abbreviations: NIHSS, National Institutes of Health Stroke Scale; OCSP, Oxfordshire Community Stroke Project; TACI, total anterior circulation infarct; PACI, partial anterior circulation infarct; POCI, posterior circulation infarct; LACI, lacunar circulation infarcts; TOAST, Trial of ORG 10172 in Acute Stroke Treatment; LAA, Large-arteryatherothrombotic; CE, Cardioembolic; SAO, Small-arteryocclusion; ODE, Other determined etiology; UDE, Undetermined etiology; BMI, body mass index.

Table S3. Univariate logistic regression for mRS trajectories of AIS patients

Variables	Moderate vs Mild		Severe vs Mild	
	OR (95% CI)	*P* value	OR (95% CI)	*P* value
Age	1.00 (0.97, 1.03)	0.992	1.06 (1.02,1.10)	**0.002**
Sex (male)	2.16 (1.10, 4.22)	**0.025**	2.20 (1.09, 4.47)	**0.029**
Marital status (Married)	0.52 (0.21, 1.32)	0.169	0.25 (0.10, 0.61)	**0.002**
Educational level				
Illiterate	1.06 (0.32, 3.52)	0.930	1.06 (0.28, 4.01)	0.937
Primary school	1.48 (0.51, 4.32)	0.472	1.38 (0.42, 4.54)	0.594
Middle school	1.00 (0.40, 2.55)	0.995	1.65 (0.63, 4.37)	0.311
High school	0.89 (0.36, 2.19)	0.798	1.05 (0.39, 2.79)	0.925
University/college or above (ref)				
Residence (Urban)	0.52 (0.21, 1.32)	0.169	0.82 (0.29, 2.32)	0.702
Occupational status				
Employed	0.49 (1.47, 1.64)	0.248	0.84 (0.20, 3.46)	0.809
Retired	0.60 (0.23, 1.56)	0.292	1.26 (0.40, 4.01)	0.692
Unemployed (ref)				
Household income				
Under 50,000 yuan	1.30 (0.50, 3.35)	0.591	1.45 (0.55, 3.84)	0.458
Among 50,000-100,000 yuan	0.60 (0.26, 1.40)	0.240	0.55 (0.22, 1.34)	0.187
Over 100,000 yuan (ref)				
NIHSS	1.70 (1.47, 1.96)	**＜0.001**	1.79 (1.55, 2.08)	**＜0.001**
OCSP classification				
TACI	3.21 (1.07, 9.66)	**0.038**	2.68 (0.72, 9.95)	0.141
PACI	2.14 (0.94, 4.85)	0.069	3.38 (1.37, 8.31)	**0.008**
POCI	2.37 (1.04, 5.38)	**0.039**	4.74 (1.97, 11.38)	**0.001**
LACI (ref)				
TOAST classification				
LAA	1.77 (0.10, 30.05)	0.695	0.68 (0.06, 8.09)	0.757
CE	0.44 (0.02, 8.04)	0.578	0.28 (0.02, 3.55)	0.327
SAO	0.33 (0.02, 5.66)	0.447	0.16 (0.01, 1.85)	0.140

ODE	1.33 (0.08, 23.54)	0.844	0.46 (0.04, 5.79)	0.547
UDE (ref)				
Therapeutic optionsn				
Intravenous thrombolysis	0.92 (0.37, 2.29)	0.850	0.46 (0.14, 1.49)	0.197
Intra-arterial thrombectomy	1.57 (0.84, 2.96)	0.160	1.81 (0.93, 3.51)	0.079
Conservative therapy (ref)				
Hypertension	2.34 (1.09, 4.99)	**0.029**	2.15 (0.98, 4.72)	0.057
Diabetes	2.60 (1.36, 4.98)	**0.004**	2.33 (1.18, 4.58)	**0.014**
Dyslipidemia	0.89 (0.38, 2.06)	0.784	1.90 (0.87, 4.14)	0.108
Atrial fibrillation	3.13 (1.25, 7.83)	**0.015**	1.63 (0.58, 4.61)	0.359
History of stroke	1.42 (0.65, 3.08)	0.381	1.60 (0.72, 3.55)	0.249
Smoking status				
Former smoker	0.83 (0.31, 2.22)	0.713	3.49 (1.18, 10.35)	**0.024**
Never smoker	0.91 (0.44, 1.89)	0.797	2.21 (0.86, 5.71)	0.101
Current smoker (ref)				
Drinking status				
Former drinker	2.87 (0.81, 10.21)	0.103	0.66 (0.22, 1.96)	0.453
Never drinker	1.57 (0.46, 5.42)	0.472	0.70 (0.26, 1.90)	0.483
Current drinker (ref)				
BMI (⩾24kg/m^2)	0.64 (0.34, 1.20)	0.160	0.55 (0.29, 1.07)	0.079
Stroke rehabilitation	7.52 (3.15, 17.93)	**＜0.001**	5.32 (2.15, 13.14)	**＜0.001**
Depressive symptoms	12.54 (5.88, 26.77)	**＜0.001**	53.25 (14.96, 189.55)	**＜0.001**

Abbreviations: mRS, modified Rankin Scale; NIHSS, National Institutes of Health Stroke Scale; OCSP, Oxfordshire Community Stroke Project; TACI, total anterior circulation infarct; PACI, partial anterior circulation infarct; POCI, posterior circulation infarct; LACI, lacunar circulation infarcts; TOAST, Trial of ORG 10172 in Acute Stroke Treatment; LAA, Large-artery atherothrombotic; CE, Cardioembolic; SAO, Small-artery occlusion; ODE, Other determined etiology; UDE, Undetermined etiology; BMI, body mass index; OR: odds ratio, CI: confidence intervals; ref, Reference. Values in bold indicate statistical significance with $P < 0.05$.

Table S4. Univariate logistic regression for BI trajectories of AIS patients

Variables	Low-rapid rise vs High-stable		Moderate low-stable vs High-stable		Moderate-progressive rise vs High-stable		Moderate high-rapid decline vs High-stable	
	OR (95% CI)	*P* value	OR (95% CI)	*P* value	OR (95% CI)	*P* value	OR (95% CI)	*P* value
Age	1.04 (0.98, 1.09)	0.187	1.03 (0.98, 1.08)	0.299	1.03 (0.99, 1.06)	0.096	1.05 (1.00, 1.11)	0.063
Sex (male)	4.05 (1.11, 14.74)	**0.034**	1.91 (0.69, 5.29)	0.217	1.33 (0.69, 2.57)	0.394	1.86 (0.61, 5.63)	0.274
Marital status (Married)	0.43 (0.13, 1.39)	0.159	0.91 (0.23, 3.52)	0.885	0.50 (0.21, 1.17)	0.109	0.37 (0.11, 1.23)	0.104
Educational level								
Illiterate	0.53 (0.051, 5.55)	0.597	1.70(0.36, 8.09)	0.505	0.99 (0.29, 3.39)	0.989	1.06 (0.08, 13.52)	0.963
Primary school	0.65 (0.10, 4.14)	0.652	0.26 (0.03, 2.52)	0.246	0.96 (0.33, 2.77)	0.938	2.62 (0.41, 16.54)	0.307
Middle school	1.57 (0.43, 5.83)	0.497	0.50 (0.12, 2.14)	0.353	0.50 (0.19, 1.33)	0.167	2.20 (0.41, 11.88)	0.358
High school	0.41 (0.08, 2.06)	0.279	0.88 (0.25, 3.11)	0.839	0.66 (0.27, 1.63)	0.365	1.10 (0.18, 6.62)	0.920
University/college or above (ref)								
Residence (Urban)	1.41 (0.29, 6.80)	0.669	0.53 (0.17, 1.69)	0.285	1.25 (0.47, 3.34)	0.652	2.66 (0.33, 21.74)	0.361
Occupational status								
Employed	1.88 (0.17, 20.61)	0.607	0.63 (0.11, 3.72)	0.606	0.44 (0.13, 1.52)	0.194	1.25 (0.10, 15.65)	0.863
Retired	2.29 (0.27, 19.16)	0.446	0.76 (0.19, 3.09)	0.703	0.66 (0.25, 1.70)	0.387	2.14 (0.26, 18.03)	0.483
Unemployed (ref)								
Household income								
Under 50,000 yuan	1.57 (0.41, 5.98)	0.512	0.70 (0.19, 2.55)	0.584	0.85 (0.33, 2.19)	0.728	6.26 (0.72, 54.41)	0.096
Among 50,000-100,000 yuan	0.49 (0.13, 1.89)	0.301	0.47 (0.15, 1.48)	0.197	0.64 (0.28, 1.48)	0.299	2.25 (0.26, 19.31)	0.461
Over 100,000 yuan (ref)								
NIHSS	2.62 (2.08, 3.30)	**＜0.001**	2.51 (2.01, 3.13)	**＜0.001**	1.83 (1.55, 2.16)	**＜0.001**	1.84 (1.51, 2.25)	**＜0.001**
OCSP classification								
TACI	7.67 (1.51, 38.98)	**0.014**	3.83 (0.75, 19.49)	0.105	3.16 (0.93, 10.74)	0.066	3.83 (0.57, 25.60)	0.165
PACI	2.00 (0.46, 8.73)	0.357	3.00 (0.95, 9.45)	0.061	1.77 (0.75, 4.15)	0.193	3.00 (0.77, 11.70)	0.114
POCI	4.38 (1.19, 16.18)	**0.027**	1.46 (0.37, 5.73)	0.587	3.09 (1.38, 6.94)	**0.006**	3.29 (0.84, 12.88)	0.088
LACI (ref)								
TOAST classification								
LAA	2.29 (0.24, 22.09)	0.475	1.05 (0.22, 5.02)	0.954	2.71 (0.49, 15.10)	0.254	0.95 (0.74, 1.22)	0.681

CE	2.00 (0.18, 22.06)	0.571	0.33 (0.04, 2.56)	0.291	2.00 (0.32, 12.51)	0.459	0.82 (0.55, 1.20)	0.306
SAO	0.14 (0.01, 2.60)	0.189	0.19 (0.03, 1.07)	0.059	1.07 (0.20, 5.90)	0.937	0.87 (0.58, 1.30)	0.500
ODE	2.40 (0.24, 24.40)	0.459	0.27 (0.04, 2.02)	0.201	3.80 (0.67, 21.60)	0.132	0.75 (0.51, 1.10)	0.138
UDE (ref)								
Therapeutic optionsn								
Intravenous thrombolysis	0.23 (0.03, 1.86)	0.169	0.85 (0.22, 3.26)	0.807	0.90 (0.39, 2.08)	0.813	0.25 (0.03,1.98)	0.188
Intra-arterial thrombectomy	1.19 (0.23, 6.26)	0.835	3.62 (1.00, 13.07)	0.051	0.85 (0.24, 3.05)	0.797	0.63 (0.07, 5.52)	0.680
Conservative therapy (ref)								
Hypertension	4.71(1.03, 21.56)	**0.046**	3.32 (0.92,12.03)	0.068	2.48 (1.14, 5.37)	**0.022**	1.83 (0.56, 6.02)	0.317
Diabetes	5.13 (1.80, 14.67)	**0.002**	1.52 (0.59, 3.95)	0.387	2.58 (1.34, 4.98)	**0.005**	1.76 (0.63, 4.91)	0.280
Dyslipidemia	2.91 (1.00, 8.49)	0.051	1.59 (0.51, 4.96)	0.426	1.14 (0.49, 2.68)	0.760	2.70 (0.88, 8.31)	0.083
Atrial fibrillation	2.15 (0.60, 7.71)	0.240	2.53 (0.77, 8.34)	0.127	1.62 (0.63, 4.16)	0.313	1.72 (0.42, 6.99)	0.448
History of stroke	1.25 (0.37, 4.23)	0.720	1.47 (0.47, 4.56)	0.504	2.00 (0.93, 4.30)	0.076	1.43 (0.42,4.91)	0.571
Smoking status								
Former smoker	1.91 (0.46, 7.92)	0.376	2.03 (0.62, 6.70)	0.244	1.27 (0.53, 3.06)	0.594	10.67 (1.22, 93.08)	0.052
Never smoker	2.05 (0.60, 7.02)	0.255	0.99 (0.31, 3.14)	0.989	1.24 (0.59, 2.61)	0.570	7.44 (0.91, 61.13)	0.062
Current smoker (ref)								
Drinking status								
Former drinker	1.45 (0.27, 7.87)	0.666	0.87 (0.19, 3.91)	0.857	1.07 (0.33, 3.43)	0.916	0.19 (0.03, 1.34)	0.097
Never drinker	0.64 (0.12, 3.53)	0.611	0.54 (0.12, 2.33)	0.405	0.94 (0.31, 2.86)	0.910	0.70 (0.17,2.94)	0.622
Current drinker (ref)								
BMI (≥24kg/m²)	0.99 (0.38, 2.61)	0.986	0.56 (0.22, 1.44)	0.229	1.16 (0.61, 2.20)	0.661	0.41 (0.14, 1.17)	0.095
Stroke rehabilitation	11.00 (3.53, 34.28)	**＜0.001**	15.89 (5.20, 48.52)	**＜0.001**	5.12 (2.09, 12.55)	**＜0.001**	5.50 (1.63, 18.60)	**＜0.001**
Depressive symptoms	38.00 (4.87, 296.71)	**0.001**	12.67 (3.49, 45.99)	**＜0.001**	2.85 (1.48,5.49)	**0.002**	34.00 (4.33, 267.02)	**0.001**

Abbreviations: BI, Barthel Index; NIHSS, National Institutes of Health Stroke Scale; OCSP, Oxfordshire Community Stroke Project; TACI, total anterior circulation infarct; PACI, partial anterior circulation infarct; POCI, posterior circulation infarct; LACI, lacunar circulation infarcts; TOAST, Trial of ORG 10172 in Acute Stroke Treatment; LAA, Large-artery atherothrombotic; CE, Cardioembolic; SAO, Small-artery occlusion; ODE, Other determined etiology; UDE, Undetermined etiology; BMI, body mass index; OR: odds ratio, CI: confidence intervals; ref, Reference. Values in bold indicate statistical significance with $P < 0.05$.

Table S5. Fit statistics of trajectory analysis

Fitting parameter	Number of group				
	2	3	4	5	6
**mRS trajectories**					
**BIC**	2288.67	2179.02	2182.74	2149.55	2183.22
**Avepp**					
Class 1	0.95	0.98	0.94	0.73	0.00
Class 2	0.95	0.93	0.75	0.99	0.63
Class 3		0.92	0.71	0.90	0.95
Class 4			0.87	0.99	0.94
Class 5				0.94	0.94
Class 6					0.92
**Class proportion, %**					
Class 1	53.42	41.55	49.14	7.31	0.00
Class 2	46.58	31.51	20.71	41.09	6.39
Class 3		26.94	16.93	24.66	36.07
Class 4			13.22	1.83	29.68
Class 5				25.11	2.74
Class 6					25.11
**BI trajectories**					
**BIC**	5468.89	5447.75	5392.36	5352.29	5381.60
**Avepp**					
Class 1	0.94	0.91	0.87	0.80	0.92
Class 2	0.96	0.95	0.93	0.89	0.85
Class 3		0.89	0.91	0.97	0.86
Class 4			0.85	0.86	0.00
Class 5				0.78	0.90
Class 6					0.94
**Class proportion, %**					
Class 1	29.22	31.8	13.61	28.77	9.59

Class 2	70.78	45.4	55.46	9.13	13.70
Class 3		22.8	7.83	43.84	29.22
Class 4			23.10	8.22	0.00
Class 5				10.05	45.21
Class 6					2.28

Abbreviations: BIC, Bayesian information criterion; AIC, Akaike’s information criterion.

Table S6. E-values for the effect of early depressive symptoms on trajectories of functional recovery in fully adjusted models

Trajectory group	OR (95% CI)	E-value estimate	E-value LL of the CI	E-value UL of the CI
**mRS Trajectories**				
Moderate vs Mild	**8.22 (2.77, 24.39)**	5.18	2.72	
Severe vs Mild	**24.41 (5.33, 111.90)**	9.35	4.05	
**BI Trajectories**				
Low-rapid rise vs High-stable	8.55 (0.75, 97.47)	5.30	1.00	
Moderate low-stable vs High-stable	2.59 (0.47, 14.36)	2.60	1.00	
Moderate-progressive rise vs High-stable	0.94 (0.36, 2.46)	1.21		1.00
Moderate high-rapid decline vs High-stable	**12.93 (1.49, 112.42)**	6.65	1.74	

Note: Fully adjusted model for mRS Trajectories: NIHSS, stroke rehabilitation, OCSP classification, hypertension, diabetes, atrial fibrillation, smoking status, age, sex, marital status. Fully adjusted model for BI Trajectories: NIHSS, Stroke rehabilitation, OCSP classification, hypertension, diabetes, sex. Abbreviations: OR, odds ratio; CI, confidence interval; LL, Lower limit of the CI; UL, Upper limit of the CI; Data with *P* values below 0.05 are presented in bold type.

Table S7. Association between early depressive symptoms and BI trajectory groups after excluding the “moderate high–rapid decline” subgroup: results of the multinomial logistic regression analysis

	class 1 vs 2		class 3 vs 2	
	OR (95% CI)	P value	OR (95% CI)	P value
Depressive symptoms	3.62 (1.36-9.67)	**0.010**	4.70 (0.90-24.69)	0.067
NIHSS	1.47 (1.25-1.72)	<0.001	2.09 (1.66-2.63)	<0.001
Stroke rehabilitation	2.75 (0.92-8.17)	0.069	2.74 (0.66-11.37)	0.166
Hypertension	0.52 (0.15-1.79)	0.303	1.05 (0.16-7.07)	0.962
Diabetes	2.95 (1.09-8.00)	0.034	3.23 (0.81-12.87)	0.096
OCSP classification				
TACI	1.11 (0.21-5.76)	0.899	1.44 (0.17-12.43)	0.743
PACI	2.57 (0.51-12.89)	0.250	3.6 (0.45-28.87)	0.228
POCI	1.36 (0.26-7.23)	0.715	2.05 (0.23-18.05)	0.518
LACI (ref)				
Sex (Male)	1.03 (0.37-2.86)	0.962	3.59 (0.70-18.04)	0.125

Abbreviations: NIHSS, National Institutes of Health Stroke Scale; OCSP, Oxfordshire Community Stroke Project; TACI, total anterior circulation infarct; PACI, partial anterior circulation infarct; POCI, posterior circulation infarct; LACI, lacunar circulation infarcts; OR: odds ratio, CI: confidence intervals; ref, Reference. Note：Class 1: moderate-low functional recovery; Class 2: high-stability; Class 3: gradual improvement; Values in bold indicate statistical significance with $P < 0.05$.

2 Supplementary Figures

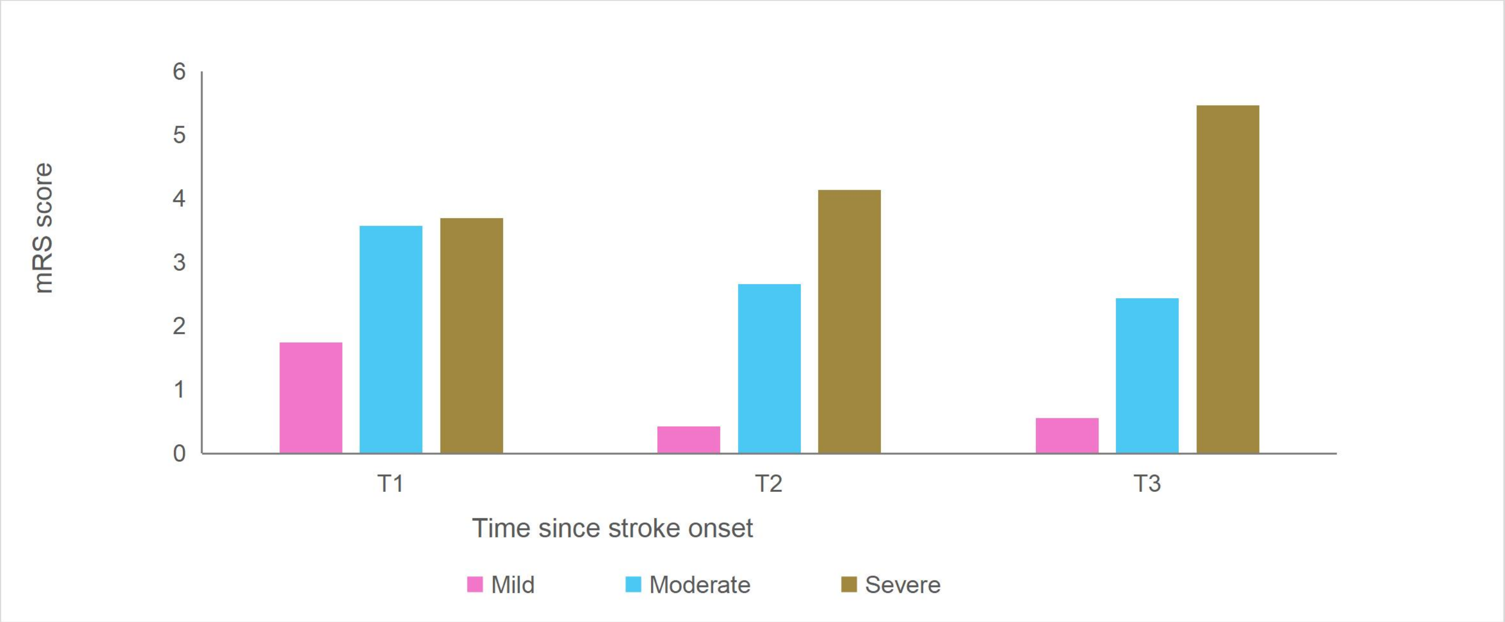

Figure S1. Functional changes at different time points by mRS trajectory groups.
Abbreviations: mRS, modified Rankin Scale; T1:baseline; T2: 3months; T3: 6 months.

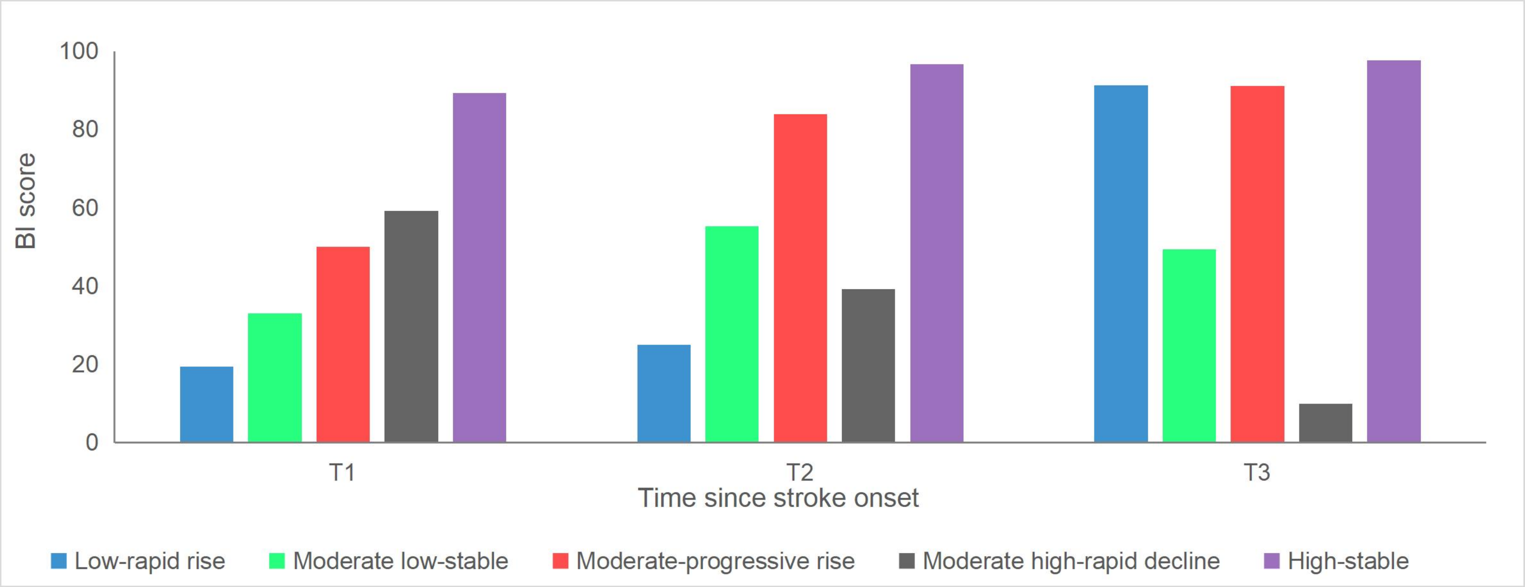

Figure S2. Functional changes across different time points by BI trajectory groups.
Abbreviations: BI, Barthel Index; T1:baseline; T2: 3 months; T3: 6 months.

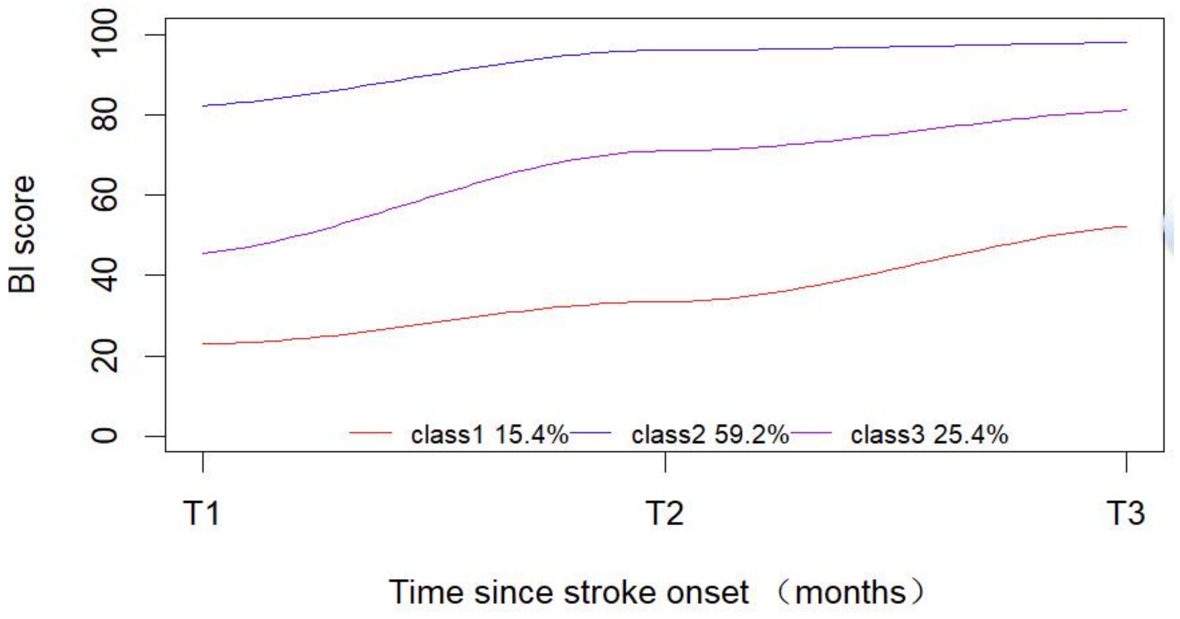

Figure S3. Trajectories of Barthel Index (BI) after excluding the “moderate high–rapid decline” subgroup: results of the sensitivity analysis in patients with acute ischemic stroke (AIS).
Class 1: moderate-low functional recovery; Class 2: high-stability; Class 3: gradual improvement;
T1: baseline; T2: 3months; T3: 6 months.
